# Gut microbiota depletion aggravates bile acid-induced liver pathology in mice with a human-like bile acid composition

**DOI:** 10.1042/CS20230812

**Published:** 2023-11-10

**Authors:** Esther Verkade, Wenqiang Shen, Milaine V. Hovingh, Niels L. Mulder, Krisztina de Bruyn, Martijn Koehorst, Hilde D. de Vries, Vincent W. Bloks, Folkert Kuipers, Jan Freark de Boer

**Affiliations:** 1 Department of Pediatrics, University of Groningen, University Medical Center Groningen, Groningen, The Netherlands; 2 Department of Laboratory Medicine, University of Groningen, University Medical Center Groningen, Groningen, The Netherlands; 3 European Research Institute for the Biology of Ageing (ERIBA), University of Groningen, University Medical Center Groningen, Groningen, the Netherlands

**Keywords:** bile acids, Cyp2c70, gut microbiota, hepatobiliary disease, hydrophobicity, liver fibrosis

## Abstract

*Cyp2c70*-deficient mice have a human-like bile acid (BA) composition due to their inability to convert chenodeoxycholic acid (CDCA) into rodent-specific muricholic acids (MCAs). However, the hydrophobic BA composition in these animals is associated with liver pathology. Although *Cyp2c70*-ablation has been shown to alter gut microbiome composition, the impact of gut bacteria on liver pathology in *Cyp2c70*^−/−^ mice remains to be established. Therefore, we treated young-adult male and female wild-type (WT) and *Cyp2c70*^−/−^ mice with antibiotics (AB) with broad specificity to deplete the gut microbiota and assessed the consequences on BA metabolism and liver pathology. Female *Cyp2c70*^−/−^ mice did not tolerate AB treatment, necessitating premature termination of the experiment. Male *Cyp2c70*^−/−^ mice did tolerate AB but showed markedly augmented liver pathology after 6 weeks of treatment. Dramatic downregulation of hepatic *Cyp8b1* expression (−99%) caused a reduction in the proportions of 12α-hydroxylated BAs in the circulating BA pools of AB-treated male *Cyp2c70*^−/−^ mice. Interestingly, the resulting increased BA hydrophobicity strongly correlated with various indicators of liver pathology. Moreover, genetic inactivation of *Cyp8b1* in livers of male *Cyp2c70*^−/−^ mice increased liver pathology, while addition of ursodeoxycholic acid to the diet prevented weight loss and liver pathology in AB-treated female *Cyp2c70*^−/−^ mice. In conclusion, depletion of gut microbiota in *Cyp2c70*^−/−^ mice aggravates liver pathology at least in part by increasing the hydrophobicity of the circulating BA pool. These findings highlight that the potential implications of AB administration to cholestatic patients should be evaluated in a systematic manner.

## Introduction

Bile acids (BAs) are cholesterol-derived amphipathic molecules that are synthesized in the liver and facilitate the absorption of dietary lipids and fat-soluble vitamins in the intestine. In addition, BAs are able to activate BA receptors in various tissues and thereby impact metabolic as well as inflammatory processes [1–6]. The primary BAs produced by the liver, cholic acid (CA) and chenodeoxycholic acid (CDCA), can be modified by gut bacteria, generating secondary BA species with distinct physicochemical properties. These secondary BAs are formed by 7α-dehydroxylation, generating deoxycholic acid (DCA) from CA and lithocholic acid (LCA) from CDCA, as well as by epimerization reactions that generate ursodeoxycholic acid (UDCA) from CDCA. Hence, the microbiome plays an important role in shaping the composition of the circulating BA pool.

For decades, translation of preclinical BA research in rodents has been complicated by the mouse-/rat-specific synthesis of very hydrophilic muricholic acids (MCAs) from hydrophobic CDCA, a reaction that does not take place in humans. Consequently, the physicochemical characteristics of the circulating BA pool differ considerably between mice and humans [7,8]. After identification of CYP2C70 as the enzyme responsible for the generation of MCAs in mice [9,10], *Cyp2c70*^−/−^ mice have been generated that have a human-like BA composition devoid of MCAs [11–13]. The hydrophobic BA composition in these mice is associated with liver pathology, which is transient in males but has a progressive nature in female *Cyp2c70*^−/−^ mice [11]. Treatment with UDCA was demonstrated to restore normal liver physiology in female *Cyp2c70*^−/−^ mice fed a regular chow diet [11], suggesting that (possibly microbiome-mediated) changes in the composition of the circulating BA pool may influence liver function. In humans, UDCA is exclusively a secondary BA, i.e., produced by gut the microbiota. However, the CYP2C70 enzyme in the liver of mice was shown to convert CDCA into UDCA [10]. Therefore, UDCA can be a primary as well as a secondary BA in these animals.

Limited information is available concerning the effects of antibiotics (AB) in the various human cholestatic liver diseases. Although data from individual studies is not conclusive, antimicrobial agents, particularly vancomycin, may ameliorate liver pathology in adults with primary sclerosing cholangitis (PSC) to some extent [14]. However, vancomycin failed to improve outcomes in pediatric PSC patients [15]. AB may impact liver pathology directly, but also indirectly. AB represent the predominant cause of drug-induced liver injury (DILI) [16,17] and mutations in the canalicular BA transporter ABCB11 as well as the canalicular phospholipid transporter ABCB4 appear to be associated with an increased risk of DILI [17]. The risk of DILI may be related to the hepatic metabolism of orally administered drugs and to whether or not they are secreted via the biliary system [18]. However, AB-induced alterations in the gut microbiome will impact BA metabolism [19] and AB may thereby affect liver pathology in an indirect manner, especially in conditions in which the liver is already prone to BA-induced injury.

In the present study, we treated *Cyp2c70*^−/−^ mice with a mixture of non-absorbable broad spectrum AB to assess the impact of gut microbiota depletion on BA metabolism and liver pathology in the context of a human-like BA composition. Surprisingly, female *Cyp2c70*^−/−^ mice did not tolerate AB treatment. AB effectively prevented the production of hydrophobic secondary BA species. Nevertheless, the overall hydrophobicity of the circulating BA pool actually increased in AB-treated male *Cyp2c70*^−/−^ mice as a result of substantially reduced CYP8B1-mediated 12α-hydroxylation of BAs during the synthesis cascade, that translated into high abundances of hydrophobic CDCA in these animals. The increased hydrophobicity of the BA pool in AB-treated male mice was associated with augmented liver pathology, characterized by increased plasma transaminases as well as severe fibrosis. Subsequent experiments demonstrated that inactivation of the *Cyp8b1* gene in the liver was sufficient to exacerbate liver pathology in male *Cyp2c70*^−/−^ mice, while the hydrophilic BA UDCA could prevent the AB-induced aggravation of liver pathology in female *Cyp2c70*^−/−^ mice. Together, these results highlight a key role for BA hydrophobicity in the aggravation of liver injury in *Cyp2c70*^−/−^ mice upon AB treatment.

## Methods

### Animals

Male and female *Cyp2c70*^−/−^ mice (C57BL/6J-Cyp2c70^em3Umcg^) [11] and wild-type (WT) littermates (15-21 weeks old) were obtained from our local breeding colony at the central animal facility of the University Medical Center Groningen. During the experiments, mice were housed under climate-controlled conditions (21°C) with a 12 h light/12 h dark cycle and had *ad libitum* access to food and drinking water. Mice were fed a regular chow diet chow (Ssniff 1554 R/M-H maintenance diet) supplemented with a mixture of the non-absorbable AB Bacitracin (Sigma-Aldrich, St. Louis, MO, U.S.A.), Neomycin (Sigma) and Vancomycin (Hikma Pharmaceuticals, London, U.K.) at a dose of 2 mg/day each for 6 weeks when indicated. A separate cohort of female 8-week-old *Cyp2c70*^−/−^ mice was pretreated with a diet containing 0.1% (w/w) UDCA (Sigma) for 2 weeks before they were switched to the AB-containing diet, either with or without UDCA, for six additional weeks. In addition, *Cyp2c70*^−/−^ mice were crossed with liver-specific Cas9-transgenic (L-Cas9_tg_) mice on a C57BL/6J background [20]. Male 8- to 10-week-old *Cyp2c70*^−/−^/L-Cas9_tg_ were injected with a self-complementary adeno-associated virus (scAAV) encoding single-guide (sg)RNAs targeting the *Cyp8b1* gene sequence, to inactivate *Cyp8b1* in hepatocytes by CRISPR/Cas9-mediated somatic genome editing, or with a control virus as detailed below. The mice were sacrificed 8 weeks after scAAV injection. All mice were fasted for 4 h (08:00–12:00 AM) prior to sacrifice by cardiac puncture under deep isoflurane-induced anesthesia. Blood was centrifuged at 8000 rpm for 10 min at 4°C and plasma was stored at −80°C until analysis. Organs were rapidly excised and snap frozen in liquid nitrogen. All animal experiments were performed at the Central Animal Facility of the University Medical Center Groningen. The animal experiments described in the present study were reviewed and approved by the Dutch national committee for animal experiments (AVD10500202115447) and experimental procedures were approved by the responsible Animal Welfare Body of the University Medical Center Groningen.

### Virus production

A DNA construct comprising an expression cassette encoding 3 sgRNA sequences directed against the *Cyp8b1* gene sequence (5′-CCTTATCTCCGCGACGGATCTGG-3′; 5′-GAGCCACCTTATCTCCGCGACGG-3′; 5′-CAAGAGTACCAGATCCGTCGCGG-3′) was synthesized (Thermo Fisher, Waltham, MA) and cloned into an scAAV2 backbone vector using KpnI and NotI (New England Biolabs, Ipswich, MA). Following sequence verification, this vector, as well as a helper plasmid (pAd-deltaF6, #112867, Addgene, Teddington, U.K.; a gift from James M. Wilson) and a Rep/Cap-encoding plasmid (pAAV2/8, #112864, Addgene; a gift from James M. Wilson) were transfected into Hek293T cells, having the adenovirus early region 1 (E1) encoded within their genome, using polyethylenimine (PEI, 23966-2, Polysciences Inc., Hirschberg an der Bergstrasse, Germany). The virus particles produced by the cells were then isolated using an iodixanol gradient and concentrated using spin filter centrifugation with a 100 kD cut-off (UFC910024, Merck, Darmstadt, Germany). The number of AAV genome copies (gc) present in the isolate was quantified using qPCR and virus aliquots were stored at −80°C until use. Mice were injected with 1 × 10^11^ gc/mouse in phosphate-buffered saline (PBS).

### Bile acid measurements

Feces was collected during 48 h, dried and thoroughly ground. Approximately 30 mg of each sample was used to extract BAs. Alkaline methanol was added to the feces and samples were heated for 3 h at 80°C, cooled down and subsequently ultrasonicated for 15 min. After intense mixing for an additional 15 min, the samples were centrifuged at 3000 × ***g*** for 10 min. Internal standards were then added to 25 µl of the supernatant prior to solid phase extraction of BAs using Oasis HLB Cartridges (Waters Milford, MA). BAs in plasma, bile and feces were quantified by ultra-high performance liquid chromatography with tandem mass spectrometry (UHPLC-MS/MS) using a Nexera X2 UHPLC system (Shimazu, Kyoto, Japan) that was coupled to a SCIEX QTRAP 4500 MD triple quadrupole mass spectrometer (SCIEX, Framingham, MA) and quantified using stable isotopically-labeled internal standards, essentially as described [11]. The hydrophobicity index of biliary BAs was calculated according to Heuman [21].

### Gene expression analyses

Snap frozen tissues were thoroughly ground prior to RNA isolation using TRI-Reagent (Sigma-Aldrich, St. Louis, MO). Isolated RNA was then reverse transcribed using Moloney Murine Leukemia Virus Reverse Transcriptase (Life Technologies, Bleiswijk, The Netherlands) and Random Nonamers (Sigma-Aldrich). Real-time-quantitative PCR (RT-qPCR) was performed on the generated cDNA using a QuantStudio-3 system (Applied Biosystems, Foster City, CA) using either Taqman primer-probe combinations or SYBR Green methodology. Gene expression was normalized to the housekeeping gene peptidyl-prolyl isomerase G (*Ppig*, *Cyclophilin G*) and further normalized to the mean of the respective control group. To analyze colonization of the intestine, bacterial DNA was isolated from caecal contents (QIAamp PowerFecal Pro DNA Kit, Qiagen, Germany) and quantified by qPCR [22]. Oligo’s used for (RT-)qPCR are listed in Supplementary Table S1.

### Plasma parameters

Plasma alanine aminotransferase (ALT), aspartate aminotransferase (AST) and alkaline phosphatase (ALP) were determined using a COBAS 6000 routine clinical chemistry analyzer with standard reagents (Roche Diagnostics, The Netherlands).

### Histology (including quantification)

Following excision of the livers, a part of the big lobe was immediately fixed in 4% formalin for 24 h prior to embedding in paraffin. Sections of 4 µm were stained with hematoxylin and eosin to assess general liver morphology or with Sirius Red and Fast Green to visualize fibrosis according to standard protocols. Immunohistochemical staining for cholangiocytes was performed using an anti-CK19 primary antibody (ab52625, Abcam, Cambridge, U.K.) and an appropriate peroxidase-conjugated secondary antibody. Immune complexes were visualized using 3,3′-diaminobenzidine (DAB). Images were obtained using a Hamamatsu NanoZoomer (Hamamatsu Photonics, Almere, The Netherlands). Collagen deposition and CK19-positive areas were quantified using ImageJ software [23] and expressed as percentage of the total image field.

### Statistics

Statistical comparisons between two groups were performed using the Mann–Whitney *U* test, whereas the Kruskal–Wallis *H* test followed by Conover post hoc comparisons was used to evaluate significance of differences between multiple groups using either GraphPad Prism (GraphPad Software, San Diego, CA, version 8), R (version 4.2.1) or Brightstat [24]. To assess associations between different parameters, Spearman correlation analysis was performed using the corrplot package (version 0.92) in R (version 4.2.1). *P-*values < 0.05 were considered statistically significant.

## Results

### Depletion of gut microbiota alters bile acid homeostasis

**Figure 1:**
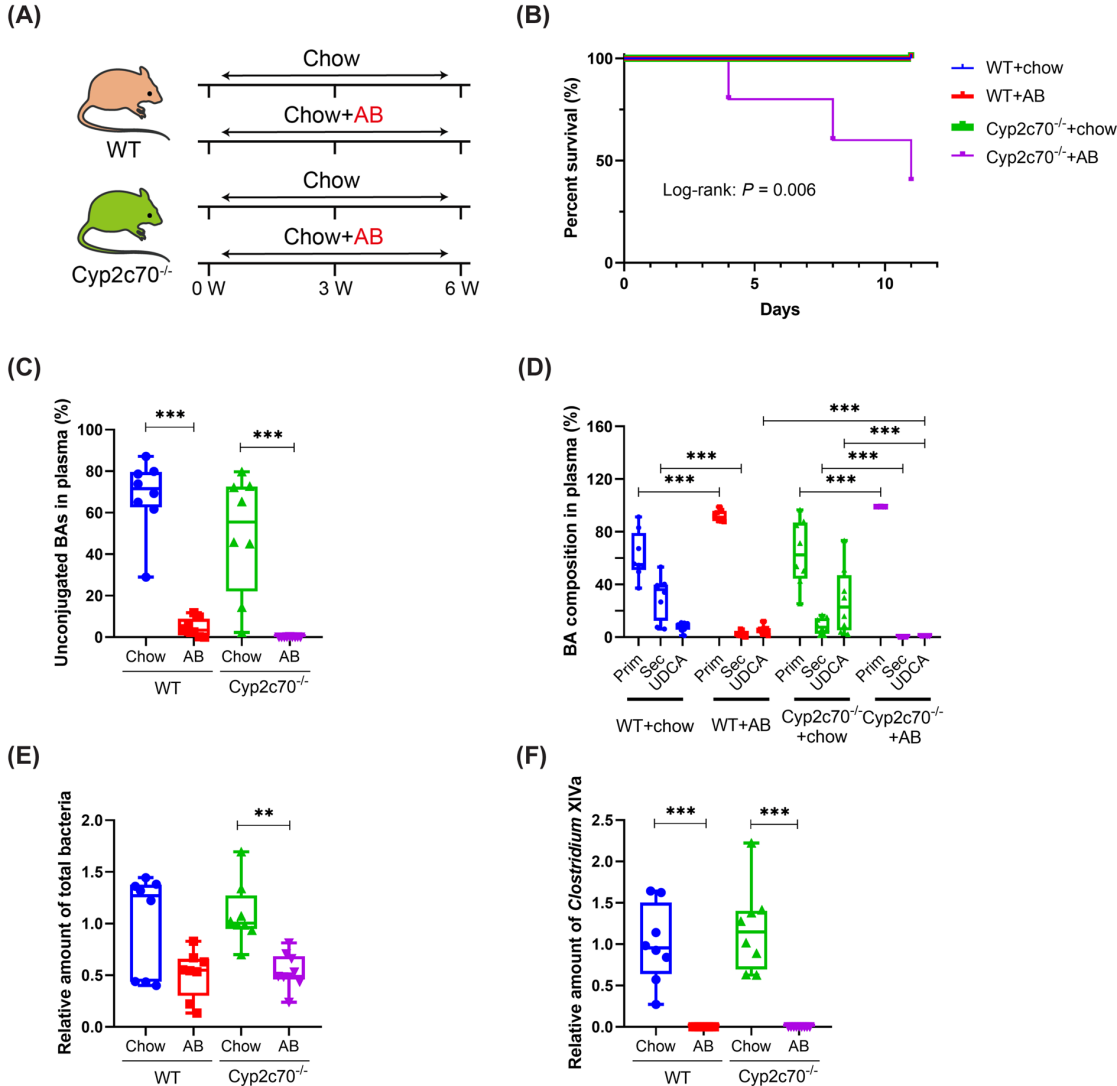
Antibiotics treatment is not tolerated by female *Cyp2c70^−/−^* mice but effectively prevents microbial bile acid deconjugation and synthesis of secondary bile acids in male mice (**A**) Study design: male (*n* = 8–9 per group) and female (*n* = 4–6 per group) WT and *Cyp2c70^−/−^* mice were fed a regular chow diet with or without AB. (**B**) Survival of female WT and *Cyp2c70^−/−^* mice over time. (**C**) Unconjugated BAs in plasma of male mice. (**D**) Percentage of primary (Prim) and secondary (Sec) BAs as well as UDCA in plasma of male mice. Primary BAs: cholic acid, chenodeoxycholic acid, α/β-muricholic acid and conjugates. Secondary BAs: deoxycholic acid, lithocholic acid, hyo(deoxy)cholic acid, ω-muricholic acid and conjugates. Relative amounts of (**E**) total bacteria and (**F**) *Clostridium* XIVa in caecum as quantified by qPCR for 16S DNA. **P<0.01, ***P<0.001 according to Kruskal–Wallis *H* test followed by Conover post-hoc comparisons. AB, antibiotics; BA, bile acid.

In order to effectively deplete their microbiota and prevent secondary BA synthesis, WT and *Cyp2c70*^−/−^ mice were treated with a mixture of Neomycin, Bacitracin and Vancomycin, which was mixed into their diet, while control animals of both genotypes received the same diet without additions (Figure 1A). AB were administered to male as well as female mice. Treatment was intended to continue for 6 weeks but, unexpectedly, female *Cyp2c70*^−/−^ mice did not tolerate AB. Substantial body weight loss and lethargy, necessitating application of humane endpoints, were frequently observed in female *Cyp2c70*^−/−^ mice on AB (Figure 1B and Supplementary Figure S1). Consequently, the experiment in female mice was terminated prematurely. In male *Cyp2c70*^−/−^ mice, severe body weight loss and lethargy were not observed during the intervention period. Therefore, only data obtained from the male mice included in this experiment are reported.

Absence of gut bacteria prevents deconjugation of primary BAs, a prerequisite for further metabolism, as well as the formation of secondary BA species. As expected, unconjugated as well as secondary BAs in the circulation were dramatically reduced in mice that received AB (Figure 1C,D and Supplementary Table S2), supporting efficient depletion of the gut microbiota. Indeed, AB treatment reduced total amounts of bacteria present in the intestines of WT and *Cyp2c70*^−/−^ mice and eliminated bacteria belonging to the *Clostridium* cluster XIVa, which are renowned secondary BA producers (Figure 1E,F). In line with the previously described capability of CYP2C70 to produce UDCA from CDCA [10], UDCA was still present in discernible amounts in AB-treated WT mice. In *Cyp2c70*^−/−^ mice, however, virtually no UDCA was detected upon AB treatment (Figure 1D), indicating that UDCA in mice can be produced by gut bacteria and by CYP2C70, but that other endogenous murine enzymes do not produce appreciable amounts of UDCA.

**Figure 2:**
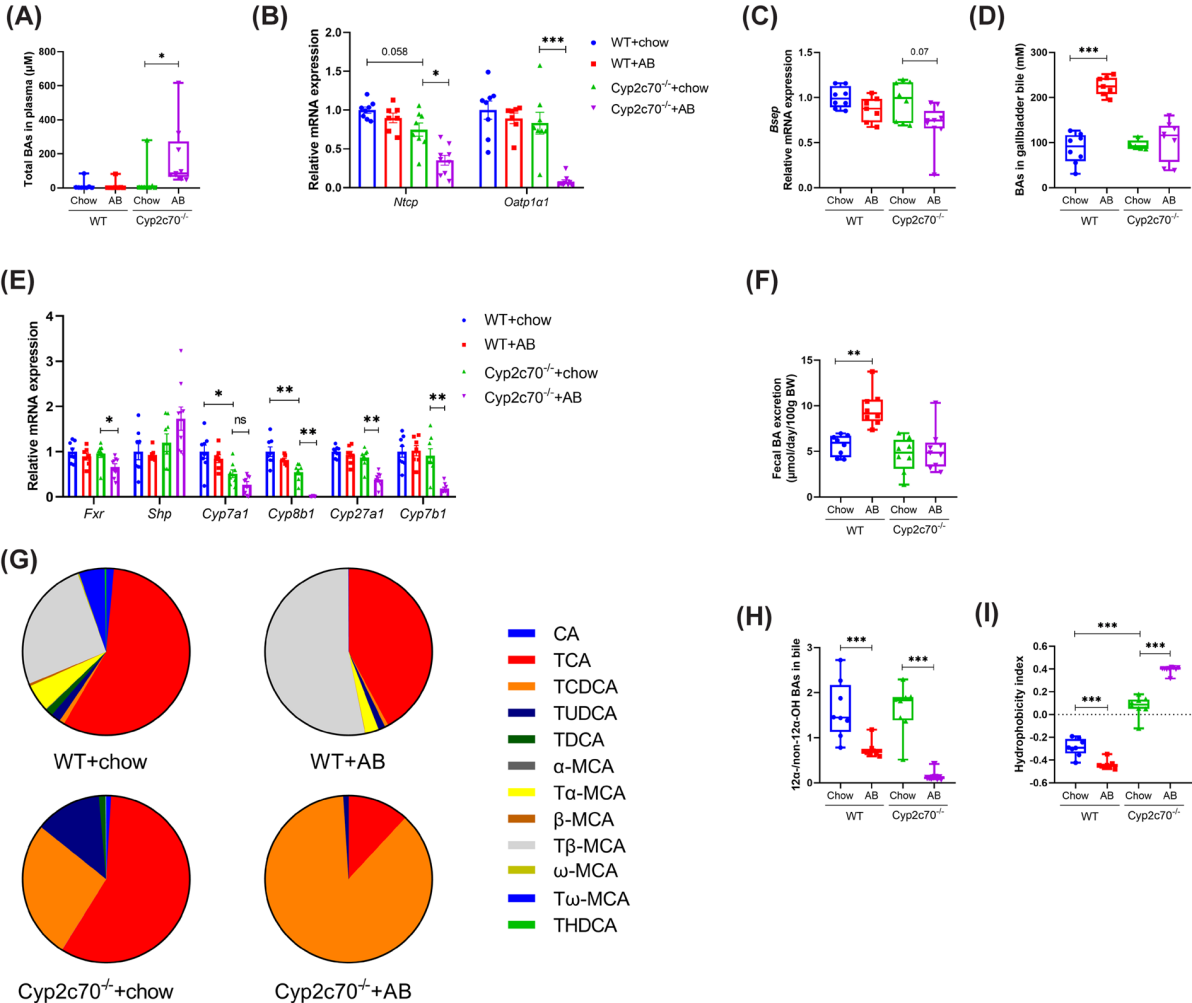
Microbiota depletion induces profound alterations in bile acid 12α-hydroxylation in male *Cyp2c70^−/−^* mice (**A**) Total BAs in plasma. (**B,C**) Relative mRNA expression of hepatic BA transporters. (**D**) Total BA concentrations in gallbladder bile. (**E**) Relative hepatic mRNA expression of genes involved in BA synthesis. (**F**) Fecal total BA excretion. (**G**) BA profiles in gallbladder bile. (**H**) 12*α*-/non-12*α-*OH BA ratios in gallbladder bile. (**I**) Hydrophobicity index of biliary BAs. *P<0.05, **P<0.01, ***P<0.001 according to Kruskal–Wallis *H-*test followed by Conover post-hoc comparisons. AB, antibiotics; BA, bile acid.

While AB did not impact total plasma BA concentrations in WT mice, BA levels in the circulation were substantially higher in *Cyp2c70*^−/−^ mice upon AB treatment (Figure 2A). Hepatic expression of the BA uptake transporters Slc10a1 (*Ntcp*) and *Slco1a1* (*Oatp1a1*) was suppressed in *Cyp2c70*^−/−^ mice receiving AB (Figure 2B) and may thus have contributed to the elevated BA levels in plasma of these animals. Expression of the bile salt export pump (*Abcb11*, *Bsep*) was, however, not significantly altered by AB treatment in WT or *Cyp2c70*^−/−^ mice (Figure 2C), while BA concentrations in gallbladder bile were considerably higher in WT mice, but remained unchanged in *Cyp2c70*^−/−^ mice upon AB treatment (Figure 2D). Hepatic gene expression analysis further revealed that expression of genes encoding the BA synthesis regulators FXR (*Nr1h4*, *Fxr*) and SHP (*Nr0b2*, *Shp*) was not affected by AB treatment in WT mice (Figure 2E). In line, expression of genes encoding key enzymes in the BA synthesis pathway remained unchanged in these mice upon AB administration. In AB-treated *Cyp2c70*^−/−^ mice, hepatic expression of *Fxr* was reduced as compared with *Cyp2c70*^−/−^ mice that did not receive AB, whereas *Shp* was not significantly affected. Furthermore, expression of the rate-controlling enzyme in BA synthesis, *Cyp7a1*, was not significantly impacted by AB administration, but expression of sterol 12α-hydroxylase, *Cyp8b1*, was dramatically decreased in *Cyp2c70*^−/−^ mice receiving AB. Expression levels of *Cyp27a1*, encoding the first enzyme in the acidic BA synthesis pathway, and *Cyp7b1*, were reduced in *Cyp2c70*^−/−^ mice upon AB treatment. Somewhat surprisingly when taking the gene expression results into account, daily fecal total BA secretion, reflecting hepatic BA synthesis during 24 h under steady state conditions, was increased in WT mice upon AB administration and remained unaffected in *Cyp2c70*^−/−^ mice (Figure 2F). Next, BA composition in feces, plasma and bile was evaluated. Fecal BAs were mostly unconjugated secondary species in WT and *Cyp2c70*^−/−^ mice that were not treated with AB whereas, in line with effective removal of gut bacteria, mostly conjugated primary BAs were present in feces of mice that had received AB (Supplementary Figure S2A–C). Interestingly, AB reduced fecal 12α-OH/non-12α-OH BA ratios in WT as well as in *Cyp2c70*^−/−^ mice (Supplementary Figure S2D). In plasma, taurocholic acid (TCA) and T-βMCA became most abundant in WT mice upon AB treatment, while non-12α-OH TCDCA accounted on average for approximately 90% of total BAs in plasma of AB-treated *Cyp2c70*^−/−^ mice (Supplementary Figure S2E,F). In bile, the relative abundance of T-βMCA was increased in WT receiving AB as compared with untreated WT mice, mainly at the expense of TCA (Figure 2G). Hence, the 12α-OH/non-12α-OH BA ratios of biliary BAs were significantly lower in WT mice that had received AB, translating into a reduced hydrophobicity index of biliary BAs, and thus into a more hydrophilic BA composition, in these animals (Figure 2H,I). In *Cyp2c70*^−/−^ mice, AB treatment resulted in a strong increase of the relative abundance of TCDCA in bile and, consequently, in strongly reduced 12α-OH/non-12α-OH BA ratios (Figure 2G,H). Importantly, and in sharp contrast to the situation in WT mice, a reduction of the 12α-OH/non-12α-OH BA ratio leads to a more hydrophobic BA composition in *Cyp2c70*^−/−^ mice (Figure 2I) due to their inability to convert hydrophobic CDCA into hydrophilic MCAs.

### Gut microbiota depletion aggravates liver pathology in *Cyp2c70*^−/−^ mice

**Figure 3:**
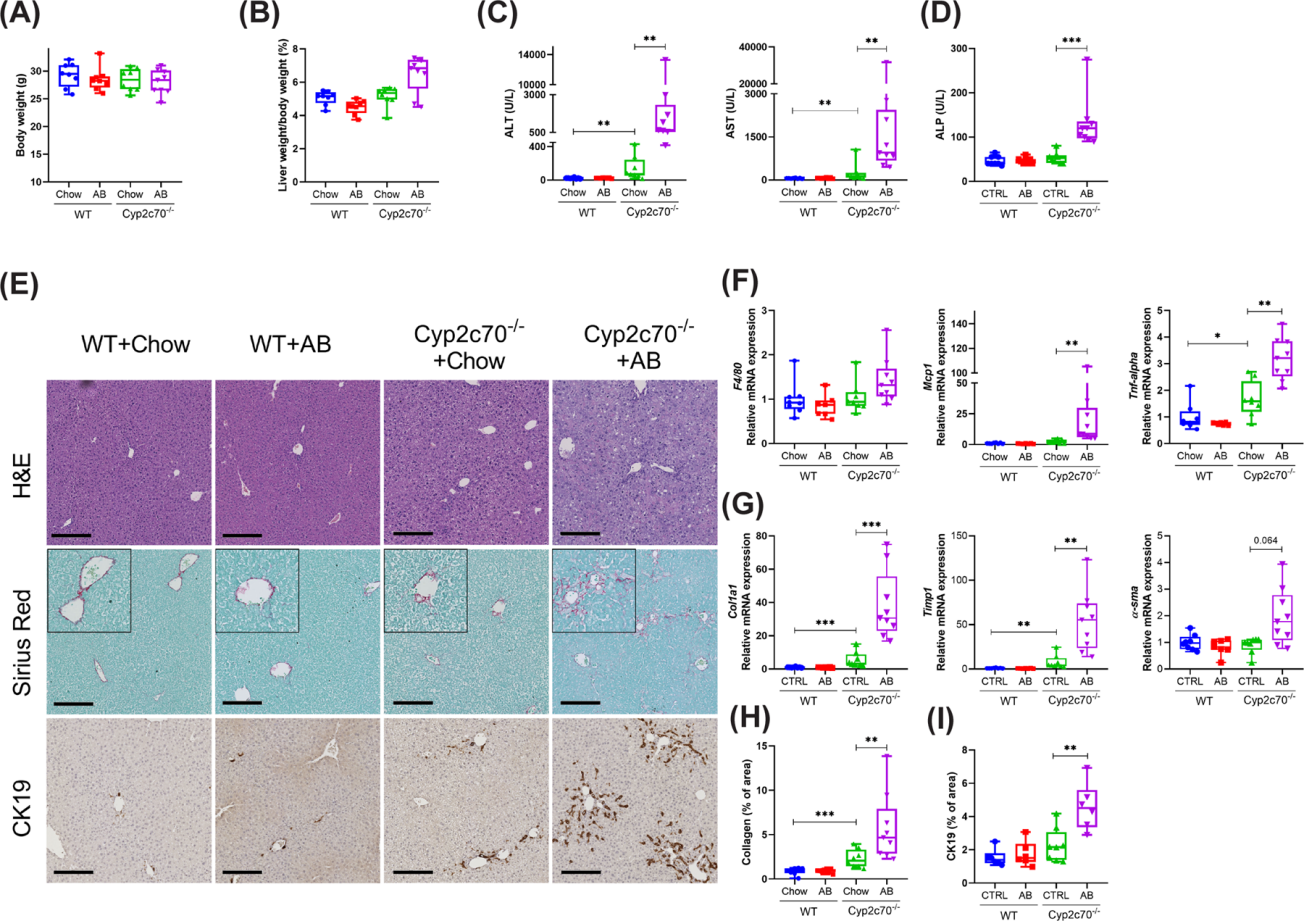
Microbiota depletion aggravates liver pathology in male *Cyp2c70^−/−^* mice (**A,B**) Body weights and relative liver weights. (**C**) Plasma transaminase and (**D**) alkaline phosphatase (ALP) levels. (**E**) Representative images of liver sections stained with hematoxylin and eosin (H&E), Sirius Red, and anti-CK19 (bars represent 200 μm). (**F,G**) Relative hepatic mRNA expression of genes involved in inflammation and fibrosis. (**H,I**) Quantification of collagen and CK19 staining, expressed as percentage positively stained area per image field. *P<0.05, **P<0.01, ***P<0.001 according to Kruskal–Wallis *H-*test followed by Conover post hoc comparisons. AB, antibiotics; CK19, cytokeratin-19.

Next, the effects of AB treatment on liver function and histology were investigated in WT and *Cyp2c70*^−/−^ mice. Body weights were similar in all groups (Figure 3A), as were liver weights of non-treated WT and *Cyp2c70*^−/−^ mice (Figure 3B). However, while AB did not impact liver weights in WT mice, AB treatment tended to increase liver weights in *Cyp2c70*^−/−^ mice (Figure 3B). Being well-established indicators of liver damage, plasma transaminases were analyzed in samples obtained after 6 weeks of AB or control treatment. Alanine aminotransferase (ALT), aspartate aminotransferase (AST) and alkaline phosphatase (ALP) were not impacted by AB in WT mice, but were substantially increased in *Cyp2c70*^−/−^ mice upon AB treatment (Figure 3C,D). Hematoxylin and eosin staining revealed frequent hepatocyte cell death in *Cyp2c70*^−/−^ mice upon AB treatment (Figure 3E and Supplementary Figure S3). Furthermore, mRNA expression of *F4/80* (*Adgre1*) did not indicate augmented macrophage infiltration in livers of AB-treated *Cyp2c70*^−/−^ mice, although increased expression of monocyte chemoattractant protein-1 (*Ccl2*, *Mcp-1*) and tumor necrosis factor α (*Tnf-alpha*) do suggest upregulation of inflammatory pathways in livers of *Cyp2c70*^−/−^ mice upon AB treatment (Figure 3F). Importantly, AB treatment substantially aggravated fibrosis and ductular reactions in *Cyp2c70*^−/−^ but not in WT mice as indicated by Sirius Red and anti-CK19 staining, respectively (Figure 3E,G–I).

**Figure 4:**
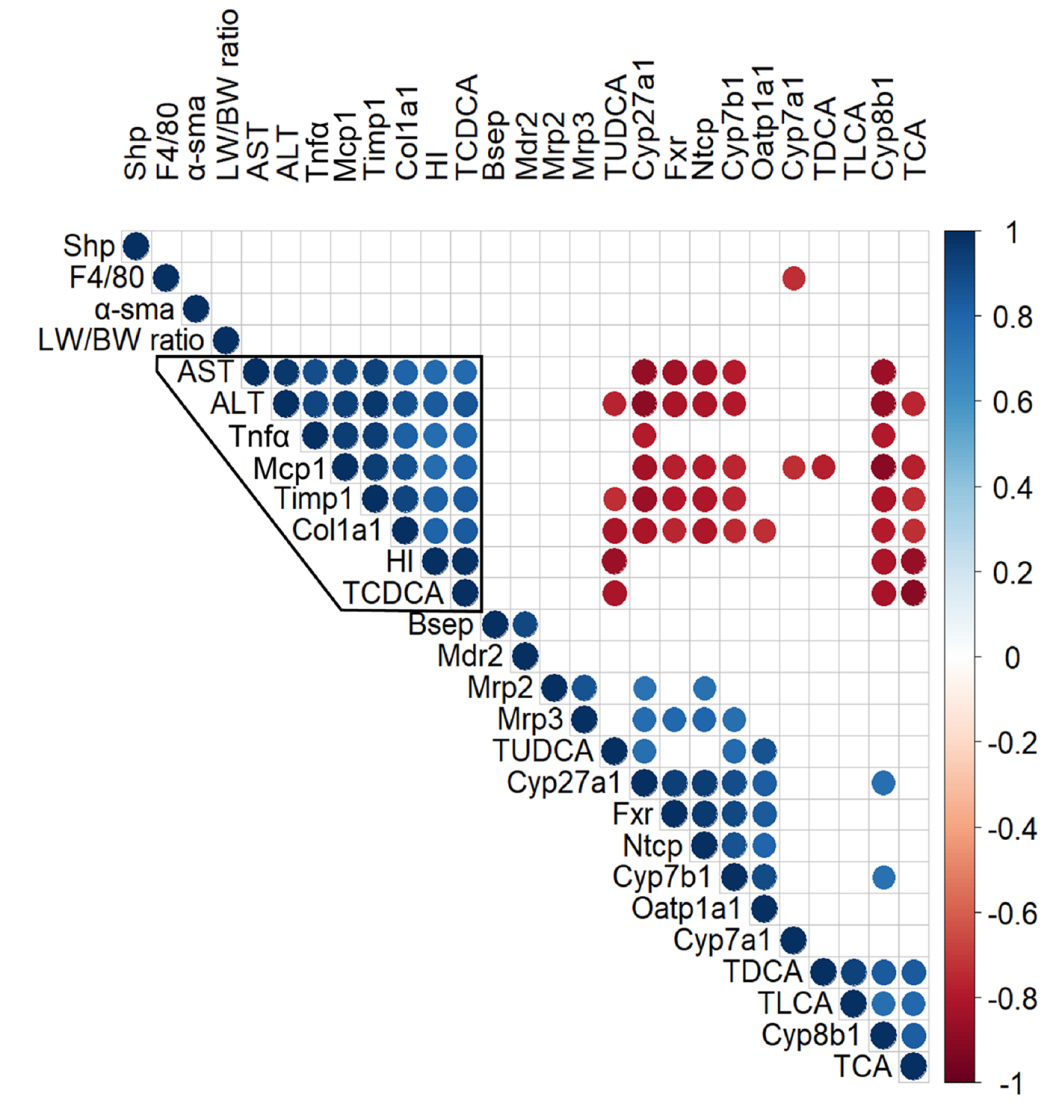
The hydrophobicity index of biliary bile acids is strongly associated with liver pathology, while *Cyp8b1* mRNA expression levels negatively correlate with indicators of liver pathology Spearman's rank-order correlation coefficients (r) were used to assess correlations between variables. Dots are displayed only for correlations with p<0.001. BW, body weight; HI, hydrophobicity index of biliary bile acids; LW, liver weight.

In an attempt to shed light on potential mechanisms contributing to the AB-induced liver phenotype of *Cyp2c70*^−/−^ mice, the strongest associations between indicators of liver pathology and parameters of BA metabolism and transport were evaluated (Figure 4). Spearman correlation analysis revealed that the hydrophobicity index of biliary BAs and the relative abundance of TCDCA clustered with indicators of liver damage, i.e., ALT, AST and expression levels of genes involved in fibrogenesis and inflammation because these parameters strongly positively correlated. Hepatic expression of *Cyp8b1* and the relative abundance of TCA in bile, as percentage of total BAs present in bile, negatively correlated with indicators of liver pathology. These data suggested a pivotal role of BA hydrophobicity in the aggravation of liver damage in *Cyp2c70*^−/−^ mice upon AB treatment.

### Ablation of hepatic *Cyp8b1* expression promotes liver pathology in *Cyp2c70*^−/−^ mice

**Figure 5:**
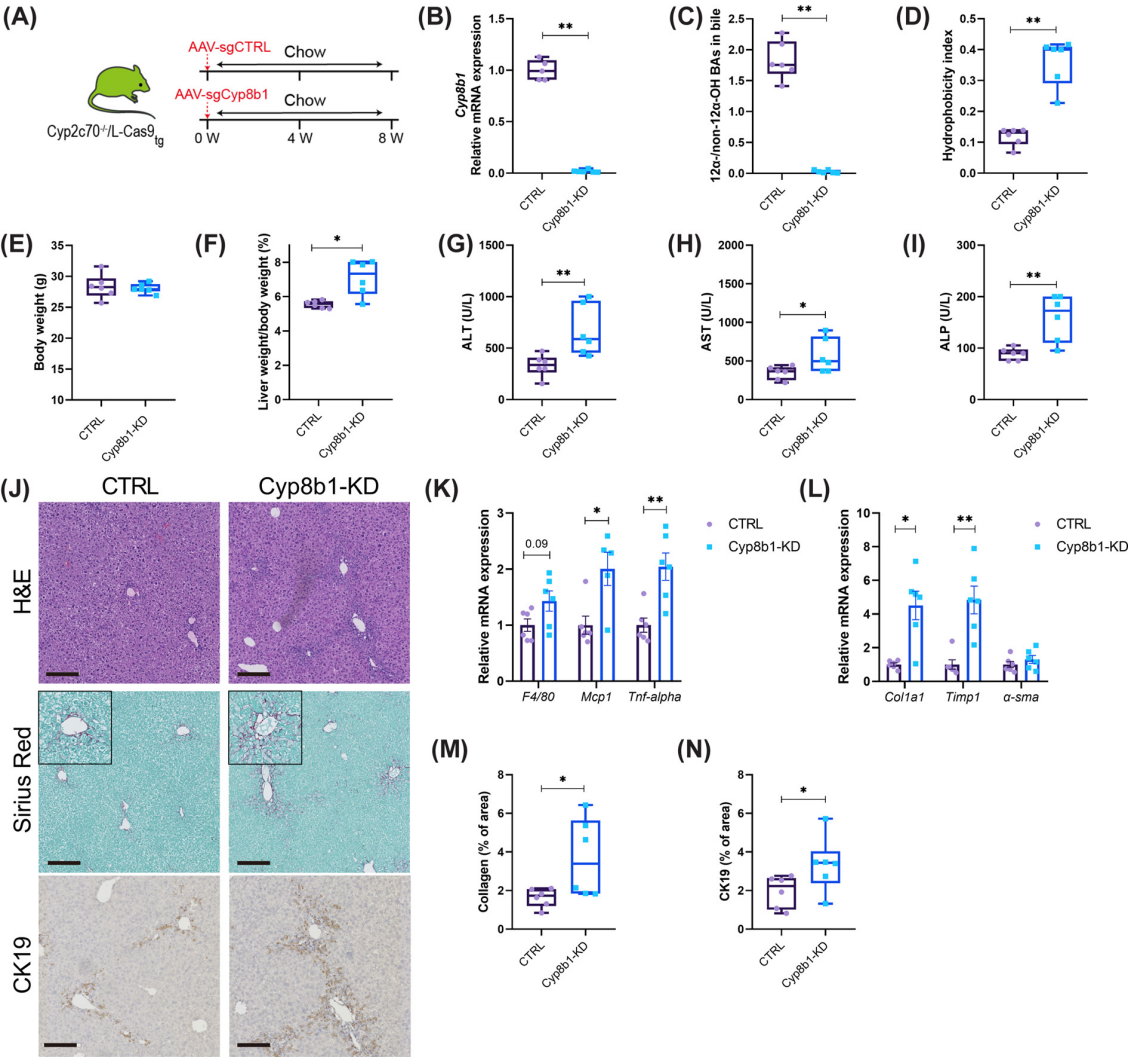
Ablation of hepatic *Cyp8b1* expression promotes liver pathology in male *Cyp2c70^−/−^* mice (**A**) Study design: male *Cyp2c70^−/−^*/L-cas9_tg_ mice (n = 6 per group) were injected with AAV-sgCTRL or AAV-sg*Cyp8b1* and maintained on a regular chow diet without additions for 8 weeks. (**B**) Relative hepatic *Cyp8b1* mRNA expression. (**C**) 12*α*-/non-12*α-*OH BA ratios in gallbladder bile. (**D**) Hydrophobicity index of biliary BAs. (**E,F**) Body weights and relative liver weights. (**G-I**) Plasma transaminase and alkaline phosphatase (ALP) levels. (**J**) Representative images of liver sections stained with hematoxylin and eosin (H&E), Sirius Red, and anti-CK19 (bars represent 200 μm). (**K,L**) Relative hepatic mRNA expression of genes involved in inflammation and fibrosis. (**M,N**) Quantification of collagen and CK19 staining, expressed as percentage positively stained area per image field. *P<0.05, **P<0.01 as determined by Mann–Whitney *U-*test. BA, bile acid; CK19, cytokeratin-19; *Cyp8b1*-KD, *Cyp8b1* knockdown.

BA hydrophobicity is to a great degree controlled by CYP8B1, which determines the balance between synthesis of hydrophobic non-12α-OH CDCA and more hydrophilic 12α-OH CA. Hence, in *Cyp2c70*^−/−^ mice, low *Cyp8b1* expression corresponded with a more hydrophobic, CDCA-enriched, composition of the circulating BA pool. To explore whether low *Cyp8b1* expression causally contributed to both BA hydrophobicity and the aggravated liver pathology observed in *Cyp2c70*^−/−^ mice upon depletion of the gut microbiota, the impact of *Cyp8b1*-knockdown (KD) on liver pathology in chow-fed *Cyp2c70*^−/−^ mice was explored. The *Cyp8b1* gene was inactivated in livers of a cohort of male *Cyp2c70*^−/−^/L-*Cas9*_tg_ mice using CRISPR/Cas9-mediated somatic genome editing technology (Figure 5A). Injection of AAV encoding sgRNAs targeting the *Cyp8b1* gene sequence efficiently reduced hepatic *Cyp8b1* expression and dramatically decreased 12α-OH BAs (Figure 5B,C; Supplementary Figure S4 and Supplementary Table S3). Consequently, the hydrophobicity of biliary BAs increased considerably (Figure 5D). Although *Cyp8b1*-KD did not impact body weight of the mice, it was associated with increased liver weight (Figure 5E,F). Furthermore, elevated plasma transaminases and ALP indicated that KD of *Cyp8b1* increased liver damage in *Cyp2c70*^−/−^ mice (Figure 5G–I), which was confirmed by liver histology. *Cyp8b1*-KD aggravated ductular reactions and activated inflammatory pathways (Figure 5J,K), which was associated with increased expression of fibrogenic genes, augmented collagen deposition as well as with cholangiocyte proliferation (Figure 5J,L–N). Taken together, these data demonstrate that low CYP8B1 activity is sufficient to promote liver pathology in mice with a human-like BA profile, i.e., *Cyp2c70*^−/−^ mice.

### Decreasing the hydrophobicity of the BA pool prevents antibiotics-induced liver pathology in *Cyp2c70*^−/−^ mice

**Figure 6:**
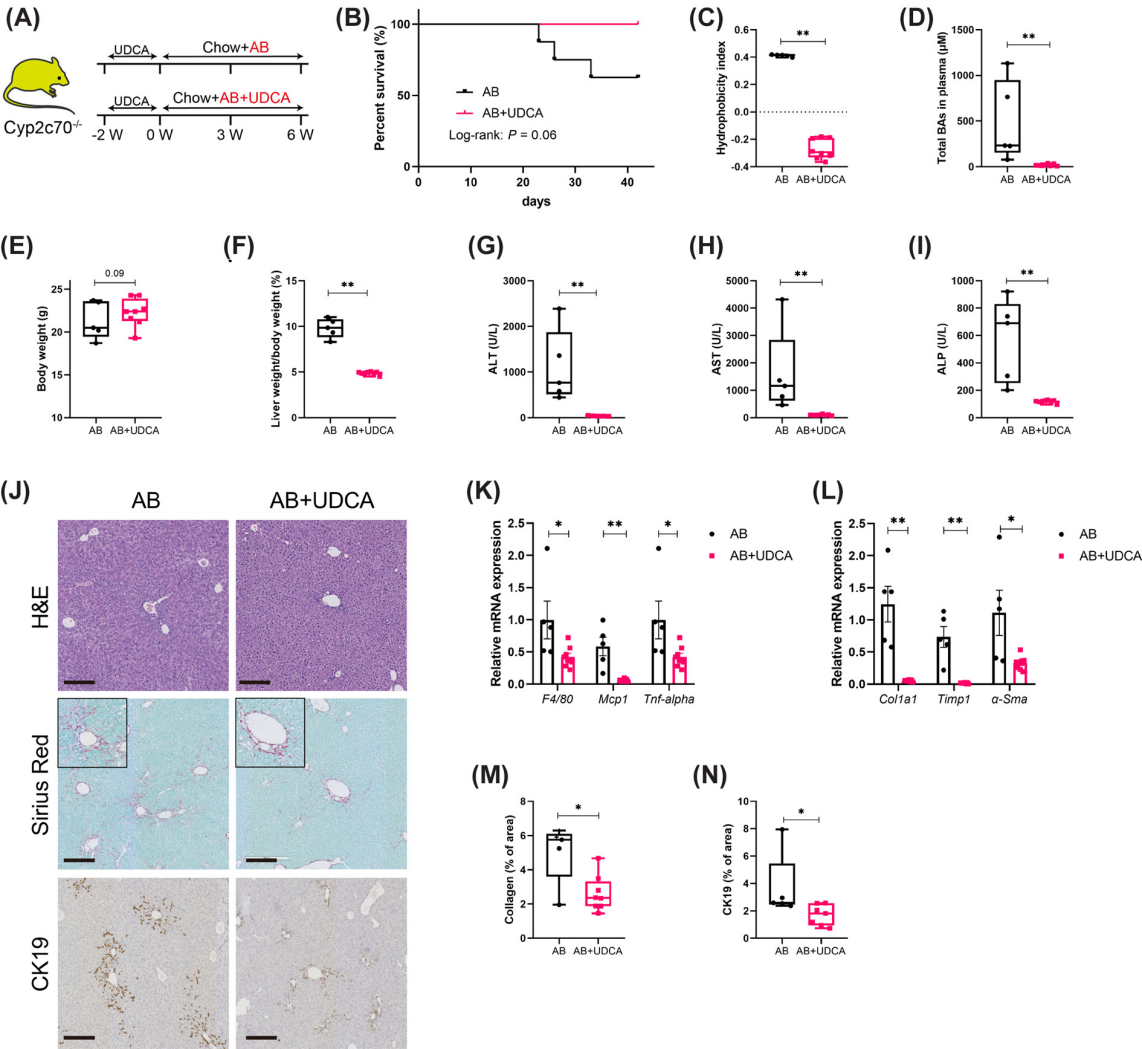
Increased hydrophilicity of the bile acid pool prevents aggravation of liver pathology upon depletion of microbiota in female Cyp2c70^−/−^ mice (**A**) Study design: female *Cyp2c70^−/−^* mice were fed a UDCA-containing diet for 2 weeks prior to the onset of AB treatment, and were then fed a diet with AB only or with AB+UDCA for 6 additional weeks (*n* = 8 mice per group). (**B**) Survival of female *Cyp2c70^−/−^* mice over time. (**C**) Hydrophobicity index of biliary BAs. (**D**) Total BAs in plasma. (**E,F**) Body weights and relative liver weights. (**G–I**) Plasma transaminase and alkaline phosphatase (ALP) levels. (**J**) Representative images of liver sections stained with hematoxylin and eosin (H&E), Sirius Red, and anti-CK19 (bars represent 200 μm). (**K,L**) Relative hepatic mRNA expression of genes involved in inflammation and fibrosis. (**M,N**) Quantification of collagen and CK19 staining, expressed as percentage positively stained area per image field. *P<0.05, **P<0.01 as determined by Mann–Whitney *U-*test. AB, antibiotics; BA, bile acid; CK19, cytokeratin-19.

To examine whether decreased hydrophobicity of the BA pool protects against the detrimental effects of microbiota depletion in *Cyp2c70*^−/−^ mice, a cohort of female mice was treated with AB as well as with the hydrophilic BA UDCA. The UDCA-containing diet was provided to all mice for 2 weeks prior to the onset of AB treatment to prevent severe AB-induced pathology before steady incorporation of the additionally supplied UDCA into the BA pool of the mice had been accomplished. After this run-in period, AB administration was started and UDCA treatment was discontinued for part of the mice, receiving AB only from that moment onwards, while UDCA administration was continued in another group of mice, receiving AB + UDCA (Figure 6A). Pre-treatment with UDCA already appeared to delay severe pathology as indicated by the prolonged survival after onset of AB treatment (compare Figures 1B and 6B). Nevertheless, humane endpoints had to be applied in 3 out of 8 mice included in the group in which UDCA treatment was discontinued at the start of AB administration, while none of the mice that kept receiving UDCA during AB treatment needed to be euthanized (Figure 6B). Analysis of the BA composition in plasma and bile confirmed that UDCA was incorporated into the circulating BA pools of the mice (Supplementary Figure S5 and Supplementary Table S4), which translated into a considerable reduction of the hydrophobicity of biliary BAs as well as into a normalization of plasma total BA concentrations (Figure 6C,D). While body weights of the mice that reached the end of the experiment did not differ significantly between the groups at the end of the experiment, liver weights were significantly lower in the mice that received UDCA in addition to AB (Figure 6E,F). Moreover, plasma levels of ALT, AST and ALP were strongly decreased in AB+UDCA-treated mice as compared to mice receiving AB only (Figure 6G–I). Liver histology was also improved in mice that received AB and UDCA as these mice showed decreased hepatocyte death and hepatic inflammation, reduced collagen deposition as well as ameliorated ductular reactions (Figure 6J–N).

Taken together, these data demonstrate that the more hydrophobic BA pool resulting from downregulation of *Cyp8b1* contributes to exacerbation of liver pathology in *Cyp2c70*^−/−^ mice upon microbiota depletion, whereas a hydrophilic BA pool preserves normal liver physiology in these mice upon AB treatment.

## Discussion

The current study reveals that depletion of the gut microbiota in the context of a human-like BA composition markedly aggravates liver pathology in adult mice and demonstrates a crucial role for BA hydrophobicity in this process. Female *Cyp2c70*^−/−^ mice did not tolerate AB-mediated depletion of their gut bacteria as indicated by severe weight loss and lethargy, while male *Cyp2c70*^−/−^ mice showed markedly augmented liver pathology but maintained normal body weight and did not become lethargic during the course of the treatment. Parameters of liver pathology strongly and positively correlated with the hydrophobicity of biliary BAs, while being negatively associated with hepatic *Cyp8b1* expression levels. In sharp contrast with WT mice, low CYP8B1 activity translates into increased BA hydrophobicity in *Cyp2c70*^−/−^ mice due to their inability to convert hydrophobic CDCA into hydrophilic α/βMCA. In line with a crucial role of BA hydrophobicity in the aggravation of liver pathology upon microbiota depletion in *Cyp2c70*^−/−^ mice, increasing BA hydrophobicity, by genetically inactivating *Cyp8b1*, by itself was sufficient to increase liver pathology in these mice, while decreasing BA hydrophobicity by treatment with the hydrophilic BA UDCA prevented hepatic inflammation, ductular reaction and fibrosis upon depletion of the microbiota by AB.

In the accompanying paper by Sjöland et al. [25], it was demonstrated that rederivatization of *Cyp2c70*^−/−^ mice as germ free (GF) was associated with severely impaired neonatal survival. Colonization of GF *Cyp2c70*^−/−^ breeding pairs with either a human or a mouse microbiota improved neonatal survival independent of donor origin. Yet, liver pathology was considerably more improved in *Cyp2c70*^−/−^ mice that had been colonized with a mouse microbiota than in those colonized with a human microbiota. Interestingly, the improved liver pathology in *Cyp2c70*^−/−^ mice colonized with a mouse microbiota was associated with increased abundances of UDCA in the circulating BA pools of these mice and, hence, with a more hydrophilic BA profile as compared with GF *Cyp2c70*^−/−^ mice and *Cyp2c70*^−/−^ mice that had been colonized with a human microbiota [25]. Whereas the study by Sjöland et al. revealed associative relationships between microbiota, BA hydrophobicity and liver pathology in *Cyp2c70*^−/−^ mice, the present study extends those observations by unequivocally demonstrating the causal role of BA hydrophobicity. We experimentally modulated the BA hydrophobicity and demonstrated that increasing the hydrophobicity (by inhibiting *Cyp8b1* expression in *Cyp2c70*^−/−^ mice) aggravated liver pathology and, reversely, decreasing BA hydrophobicity (by administration of the hydrophilic BA UDCA) prevented the development of liver pathology in AB-treated *Cyp2c70*^−/−^ mice.

In addition to inducing a more hydrophobic BA profile, microbiota depletion may also impact liver pathology in *Cyp2c70*^−/−^ mice via other mechanisms. We have previously demonstrated that depletion of gut microbiota by AB treatment translates into increased as well as more proximal expression of the apical sodium-dependent bile salt transporter (ASBT) that mediates intestinal uptake of BAs and, thus, plays a crucial role in their recirculation to the liver [19]. AB treatment in WT mice increased the biliary BA secretion rate more than 4-fold, likely related to a more proximal expression of ABST in the small intestine and thereby accelerated enterohepatic cycling of BAs [19]. An increased flux of BAs through the liver may aggravate liver damage, as illustrated by data on *Abcb4*^−/−^ mice. *Abcb4*^−/−^ mice are capable of producing hydrophilic MCAs but lack hepatoprotective biliary phospholipid secretion. Absence of a microbiota also exacerbates liver pathology in these mice [26]. As low CYP8B1 activity would translate into a more hydrophilic profile in these mice, lack of microbiota-mediated production of UDCA and/or accelerated enterohepatic cycling of BAs might have been major drivers of liver pathology in those experiments. Furthermore, in a recent study GF *Abcb4*^−/−^ mice were reported to have 100% lethality by the age of 8 weeks, while vancomycin treatment aggravated liver pathology in *Abcb4*^−/−^ mice [27]. Data on hepatic *Cyp8b1* expression, BA hydrophobicity, or detailed BA composition were, however, not provided in that study. Following bile duct ligation, GF WT C57BL/6 mice showed larger bile infarct areas within their livers as compared with bile duct-ligated controls possessing a microbiota [28]. However, microbiological status did not have an evident impact on the BA profile under these conditions. Therefore, it cannot ruled out that, besides BA hydrophobicity, other factors also contribute to exacerbation of liver pathology in *Cyp2c70*^−/−^ mice upon depletion of their microbiota.

The present study indicates an important role for CYP8B1 in the aggravation of liver pathology upon microbiota depletion in *Cyp2c70*^−/−^ mice because of its role in regulating the hydrophobicity of the circulating BA pool. The mechanism underlying the dramatic downregulation of hepatic *Cyp8b1* expression in AB-treated *Cyp2c70*^−/−^ mice remains to be determined. Although certain lineages independently lost CYP8B1 during mammalian evolution [29], the *CYP8B1* gene is conserved in humans and mice and is devoid of introns in both species [30]. In humans as well as in mice, FXR is a major regulator of BA synthesis and its activation results in repression of *Cyp7a1* as well as of *Cyp8b1*. In mice, *Cyp8b1* repression is reported to be regulated mostly by hepatic FXR, while repression by intestinal FXR via FGF15-FGFR4 may be less important [31]. In the present study, however, hepatic *Fxr* expression was slightly reduced in *Cyp2c70*^−/−^ mice upon AB treatment, while expression of its target gene *Nr0b2* (*Shp*) was not significantly altered. A major role for FXR in the strong downregulation of *Cyp8b1* upon microbiota depletion is therefore not likely. Furthermore, HNF-4α and LRH-1, which are both inhibited by SHP [32,33], induce expression of *Cyp8b1* [33,34]. In our present study, depletion of the gut microbiota did not significantly affect hepatic expression levels of *Hnf4a* or *Nr5a2*, encoding HNF-4α and LRH-1, respectively (data not shown). Although protein levels were not quantified, it is therefore not likely that these transcriptional regulators played a major role in the strong downregulation of *Cyp8b1* expression that was observed in *Cyp2c70*^−/−^ mice upon microbiota depletion in this study. Hepatic mRNA expression of the transcriptional repressor musculoaponeurotic fibrosarcoma oncogene homolog G (MAFG), that inhibits *Cyp8b1* transcription by binding to MAFG response elements (MAREs) in its gene promoter [35], also remained unchanged in *Cyp2c70*^−/−^ mice upon AB treatment (data not shown). Hence, MAFG most likely does not play a major role in the strong downregulation of *Cyp8b1* that takes place upon depletion of the gut microbiota in *Cyp2c70*^−/−^ mice. Interestingly, hepatic *CYP8B1* expression levels in PSC patients were only 36% of those observed in controls, while CYP7A1 mRNA and protein levels did not differ between both groups [36]. This observation suggests that these patients have a relatively decreased CA synthesis compared with CDCA synthesis and thus have a relatively hydrophobic BA pool. Patients with progressive familial intrahepatic cholestasis type 1 (PFIC1) have increased CDCA/CA ratios in plasma [37], indicative for a hydrophobic BA pool. It remains to be determined to what extent the composition of the BA pool impacts the natural course of disease in these patient populations, as large variations in BA composition are known to exist in individuals without apparent liver disease [38].

In conclusion, the present study demonstrates that depletion of the gut microbiota aggravates liver pathology in* Cyp2c70*^−/−^ mice as indicated by a marked elevation of liver damage markers in plasma as well as by augmented ductular reactions, activation of inflammatory gene expression pathways and increased fibrosis. This phenotype appears to be, at least in part, caused by an increased hydrophobicity of the circulating BA pool due to a sharp downregulation of hepatic *Cyp8b1*, as its genetic inactivation was sufficient to exacerbate liver pathology in *Cyp2c70*^−/−^ mice while administration of hydrophilic UDCA neutralized the detrimental effects of microbiota depletion in these mice with a human-like BA composition. The potential detrimental effects of a hydrophobic BA pool, especially in conditions in which the liver is prone to BA-induced damage, warrant that the potential implications of AB administration to patients with cholestatic liver diseases are evaluated in a comprehensive manner.

## Clinical perspectives

- Cyp2c70^-/-^ mice have a hydrophobic, human-like bile acid composition due to their inability to convert chenodeoxycholic acid into hydrophilic mouse-specific muricholic acid species and therefore represents a new, more humanized, preclinical model to study bile acid-related liver diseases. However, the influence of microbiota-mediated modulations of bile acid metabolism on liver (patho)physiology in this mouse model are unknown.
- Depletion of gut microbiota with broad spectrum antibiotics prevented the synthesis of secondary bile acids, but aggravated liver pathology in adult Cyp2c70^-/-^ mice. Our data indicate that the effects were at least in part due to impaired 12α-hydroxylation of bile acids during their synthesis, translating into a substantially more hydrophobic, and thus more hepatotoxic, bile acid composition in these mice.
- Our findings indicate that effects of the gut microbiome on bile acid metabolism need to be carefully evaluated when considering prescribtion of antibiotics to patients whose livers may be prone to develop bile acid-induced damage, such as patients with cholestatic liver diseases.

## Supplementary Material

Supplementary Figures S1-S5 and Tables S1-S4

## Data Availability

All supporting data are included within the main article and its supplementary files.

## Open Access

Open access for this article was enabled by the participation of University of Groningen in an all-inclusive *Read & Publish* agreement with Portland Press and the Biochemical Society under a transformative agreement with Individual.

## Abbreviations

AB: antibiotics
ASBT: apical sodium-dependent bile salt transporter
BA: bile acid
CA: cholic acid
CDCA: chenodeoxycholic acid
CYP2C70: cytochrome P450, family 2, subfamily c, polypeptide 70
DAB: 3,3′-diaminobenzidine
DCA: deoxycholic acid
GF: germ free
HI: hydrophobicity index
KD: knockdown
LCA: lithocholic acid
MCA: muricholic acid
TCA: taurocholic acid
UDCA: ursodeoxycholic acid
WT: wild-type
