# Supplementary material for: Gut microbiota depletion aggravates bile acid-induced liver pathology in mice with a human-like bile acid composition
Source: Clin Sci (Lond). 2023 Nov 10;137(21):1637–50. doi: 10.1042/CS20230812 (PMC10643054; doi:10.1042/CS20230812)
Supplement: Supplementary Figures S1-S5 and Tables S1-S4 [file CS-2023-0812_supp.pdf]

Supplementary data

Supplementary Table 1. Taqman and SYBR Green PCR primer and probe sequences.

Gene		**Taqmanoligo's (5’→3’)**	**Gene**		**Taqmanoligo's (5’→3’)**
	Fw	TTCGTGTGGCCCCTGAAG		Fw	TGCTGGAGTGAGCTCTTGATTC
α-sma	Rv	GGACAGCACAGCCTGAATAGC	***Lrh1***	Rv	GATGGTGGAGTAGTCCACGTGT
	Probe	TTGAGACCTTCAATGTCCCCGCCA		Probe	CCTTCCTTCCCATGCGCCACTTG
	Fw	CGGCTCCTGCTCCTCTTAGG		Fw	GGCTCAGCCAGATGCAGTTAA
Col1a1	Rv	CTGACTTCAGGGATGTCTTCTTGG	***Mcp1***	Rv	AGCCTACTCATTGGGATCATCTT
	Probe	CCACTGCCCTCCTGACGCATGG		Probe	CCCCACTCACCTGCTGCTACTCATTCA
	Fw	GCCTTGCACAAGGAAGTGACT		Fw	ATGACCACCTGCTCCAGCTT
Cyp27a1	Rv	CGCAGGGTCTCCTTAATCACA	***Ntcp***	Rv	GCCTTTGTAGGGCACCTTGT
	Probe	CCCTTCGGGAAGGTGCCCCAG		Probe	CCTTGGGCATGATGCCTCTCCTC
	Fw	CAGGGAGATGCTCTGTGTTCA		Fw	CAGTCTTACGAGTGTGCTCCAGAT
	Rv	AGGCATACATCCCTTCCGTGA		Rv	ATGAGGAATACTGCCTCTGAAGTG
Cyp7a1	Probe	TGCAAAACCTCCAATCTGTCATGA GACCTCC	***Oatp1a1***	Probe	TGGATTTGCCAGTACATTTACCTTCTTGCCC
	Fw	TGAAATAGGAGCACATCATCTTGG		Fw	CAGATCGAGGGATCGATTCAG
Cyp7b1	Rv	AATACATTGCCCAGAACATAGCTG	***Ppig***	Rv	TCACCACTTGACACCCTCATTC
	Probe	CTCTGGGCCTCTCTAGCAAACACCATTC		Probe	CTCCTCCACATTGGAGACAAGAGATGCA
	Fw	AAGGCTGGCTTCCTGAGCTT		Fw	AAGGGCACGATCCTCTTCAA
Cyp8b1	Rv	AACAGCTCATCGGCCTCATC	***Shp***	Rv	CTGTTGCAGGTGTGCGATGT
	Probe	CGGCTACACCAAGGACAAGCAGCAAG		Probe	ATGTGCCAGGCCTCCGTGCC
	Fw	TCAAGGACACGAGGTTGCTGA		Fw	TCTGAGCCCTGCTCAGCAA
F4/80	Rv	CCAAGGGGCCAATCTGGAA	***Timp1***	Rv	AACAGGGAAACACTGTGCACAC
	Probe	CCAGCACCCAGGAGCAGCCCA		Probe	CCACAGCCAGCACTATAGGTCTTTGAGAAAGC
	Fw	GAGGGCTGCAAAGGTTTCTTC		Fw	GTAGCCCACGTCGTAGCAAAC
Fxr	Rv	ACTTCCTGCGCATGTACATGTC	***Tnf-alpha***	Rv	AGTTGGTTGTCTTTGAGATCCATG
	Probe	CCGTTCTTACACTTGTACACGGCGTTCTTG		Probe	CGCTGGCTCAGCCACTCCAGC
	Fw	ATGCCAAGGGGCTGAGTGAC			
Hnf4-alpha	Rv	GCCGGTCGTTGATGTAATCCT			
	Probe	CACCTGTGACCGCAGCCGCTTG			
Gene		**SYBR Green oligo's (5’→3’)**	Gene		**SYBR Green oligo's (5’→3’)**
Ppig	Fw	GGGGATAAAGGTCCAGCGT	***Mafg***	Fw	CAAGGCCTTAAAGGTGAAGCG
	Rv	AGACAACTCTTCCAGCAGGT		Rv	TTCAACTCTCGCACCGACAT
16SrRNA	UniF340	ACTCCTACGGGAGGCAGCAGT	***Clostridium***	Fw	AAATGACGGTACCTGACTAA
	UniR514	ATTACCGCGGCTGCTGGC	**ClusterXIVa**	Rv	CTTTGAGTTTCATTCTTGCGAA

Supplementary Table 2. Plasma bile acid concentrations of male WT mice and *Cyp2c70*$^{-/-}$ mice on either chow or antibiotics. Data represents median ± interquartile range. * p< 0.05 vs. chow control of same genotype, # p < 0.05 vs. WT with same treatment according to Kruskal-Wallis H test followed by Conover post hoc comparisons. AB, antibiotics.

	Males			
**Concentration (µmol/L)**	**WT + Chow (n=8)**	**WT + AB (n=8)**	***Cyp2c70*$^{-/-}$ + Chow (n=8)**	***Cyp2c70*$^{-/-}$ + AB (n=9)**
CA	0.687 (0.123-1.383)	0.000 * (0.000-0.000)	0.545 (0.273-1.209)	0.000 * (0.000-0.0130)
TCA	0.231 (0.099-1.342)	0.474 (0.280-1.230)	0.816 (0.314-4.850)	6.800 * # (6.220-34.100)
CDCA	0.0540 (0.036-0.098)	0.000 * (0.000-0.000)	0.490 # (0.322-1.412)	0.046 * # (0.038-0.086)
TCDCA	0.026 (0.000-0.045)	0.029 (0.026-0.040)	0.456 # (0.168-3.410)	78.80 * # (55.80-237.0)
UDCA	0.164 (0.080-0.332)	0.000 * (0.000-0.000)	0.558 # (0.275-1.239)	0.026 * (0.000-0.029)
TUDCA	0.042 (0.036-0.103)	0.060 (0.042-0.075)	0.252 # (0.221-0.347)	0.792 * # (0.576-1.680)
DCA	0.212 (0.120-0.565)	0.000 * (0.000-0.000)	0.224 (0.156-0.369)	0.000 * (0.000-0.000)
TDCA	0.047 (0.038-0.172)	0.000 * (0.000-0.000)	0.129 (0.049-0.457)	0.000 * (0.000-0.000)
LCA	0.031 (0.007-0.035)	0.030 (0.000-0.032)	0.078 # (0.054-0.112)	0.000 * (0.000-0.029)
TLCA	0.000 (0.000-0.000)	0.000 (0.000-0.000)	0.030 # (0.000-0.065)	0.000 (0.000-0.033)
αMCA	0.044 (0.026-0.065)	0.000 * (0.000-0.000)	0.000 # (0.000-0.000)	0.000 (0.000-0.000)
TαMCA	0.045 (0.027-0.157)	0.051 (0.039-0.146)	0.000 # (0.000-0.000)	0.000 # (0.000-0.000)
βMCA	0.474 (0.195-1.087)	0.026 * (0.006-0.038)	0.000 # (0.000-0.000)	0.000 # (0.000-0.000)
TβMCA	0.065 (0.035-0.330)	0.274 * (0.167-1.142)	0.000 # (0.000-0.000)	0.000 # (0.000-0.000)
ωMCA	0.387 (0.200-0.857)	0.000 * (0.000-0.000)	0.000 # (0.000-0.000)	0.000 (0.000-0.000)
TωMCA	0.074 (0.059-0.170)	0.000 * (0.000-0.000)	0.000 # (0.000-0.000)	0.000 (0.000-0.000)
HDCA	0.000 (0.000-0.040)	0.000 (0.000-0.000)	0.000 (0.000-0.000)	0.000 (0.000-0.000)
THDCA	0.000 (0.000-0.000)	0.000 (0.000-0.000)	0.000 (0.000-0.000)	0.000 (0.000-0.000)
Total	2.860 (1.185-6.304)	0.926 (0.587-2.709)	3.996 (2.595-11.410)	84.680 * # (63.630-273.900)

Supplementary Table 3. Plasma bile acid concentrations of male *Cyp2c70*$^{-/-}$ mice injected with control virus or *Cyp8b1*-KD virus. Data represents median ± interquartile range. * $p < 0.05$ as determined by Mann-Whitney U test. CTRL, control; *Cyp8b1*-KD, *Cyp8b1* knockdown.

	**Males**	
**Concentration (µmol/L)**	***Cyp2c70*$^{-/-}$ CTRL (n=6)**	***Cyp2c70*$^{-/-}$ *Cyp8b1*-KD (n=5)**
CA	0.717 (0.209-0.887)	0.047 (0.013-0.653)
TCA	2.220 (0.506-10.250)	0.101 (0.019-4.021)
CDCA	1.040 (0.549-1.725)	4.280 * (3.800-17.530)
TCDCA	1.435 (0.370-5.980)	17.700 * (3.305-100.600)
UDCA	0.182 (0.065-0.956)	0.198 (0.170-1.569)
TUDCA	0.206 (0.084-0.558)	0.928 (0.409-2.395)
DCA	0.046 (0.000-0.113)	0.000 (0.000-0.067)
TDCA	0.018 (0.000-0.067)	0.000 (0.000-0.066)
LCA	0.019 (0.000-0.064)	0.394 * (0.243-0.835)
TLCA	0.000 (0.000-0.000)	0.137 * (0.089-0.997)
αMCA	0.000 (0.000-0.000)	0.000 (0.000-0.000)
TαMCA	0.000 (0.000-0.000)	0.000 (0.000-0.000)
βMCA	0.000 (0.000-0.000)	0.000 (0.000-0.000)
TβMCA	0.000 (0.000-0.000)	0.000 (0.000-0.000)
ωMCA	0.000 (0.000-0.000)	0.000 (0.000-0.000)
TωMCA	0.000 (0.000-0.000)	0.000 (0.000-0.000)
HDCA	0.000 (0.000-0.000)	0.000 (0.000-0.022)
THDCA	0.000 (0.000-0.000)	0.026 (0.000-0.194)
Total	7.163 (2.244-18.93)	22.52 (9.900-127.5)

Supplementary Table 4. Plasma bile acid concentrations of female *Cyp2c70*$^{-/-}$ mice on antibiotics with or without UDCA supplementation. Data represents median ± interquartile range. * p< 0.05 as determined by Mann-Whitney U test. AB, antibiotics; UDCA, ursodeoxycholic acid.

	**Females**	
**Concentration (µmol/L)**	***Cyp2c70*$^{-/-}$ AB (n=5)**	***Cyp2c70*$^{-/-}$ AB+UDCA (n=8)**
CA	0.000 (0.000-0.058)	0.000 (0.000-0.000)
TCA	26.500 (10.400-100.300)	0.580 * (0.345-1.713)
CDCA	0.103 (0.046-0.119)	0.000 * (0.000-0.000)
TCDCA	209.000 (135.900-827.000)	3.005 * (1.415-6.170)
UDCA	0.026 (0.000-0.0298)	0.303 * (0.164-1.740)
TUDCA	2.870 (1.421-11.140)	10.550 (5.580-17.230)
DCA	0.000 (0.000-0.000)	0.000 (0.000-0.000)
TDCA	0.000 (0.000-0.000)	0.000 (0.000-0.000)
LCA	0.000 (0.000-0.000)	0.000 (0.000-0.000)
TLCA	0.1330 (0.019-0.648)	0.000 * (0.000-0.000)
αMCA	0.000 (0.000-0.000)	0.000 (0.000-0.000)
TαMCA	0.000 (0.000-0.000)	0.000 (0.000-0.000)
βMCA	0.000 (0.000-0.000)	0.000 (0.000-0.000)
TβMCA	0.000 (0.000-0.000)	0.000 (0.000-0.000)
ωMCA	0.000 (0.000-0.000)	0.000 (0.000-0.000)
TωMCA	0.000 (0.000-0.000)	0.000 (0.000-0.000)
HDCA	0.000 (0.000-0.000)	0.000 (0.000-0.000)
THDCA	0.000 (0.000-0.000)	0.000 (0.000-0.000)
Total	231.7 (151.200-939.200)	14.930 * (9.949-28.540)

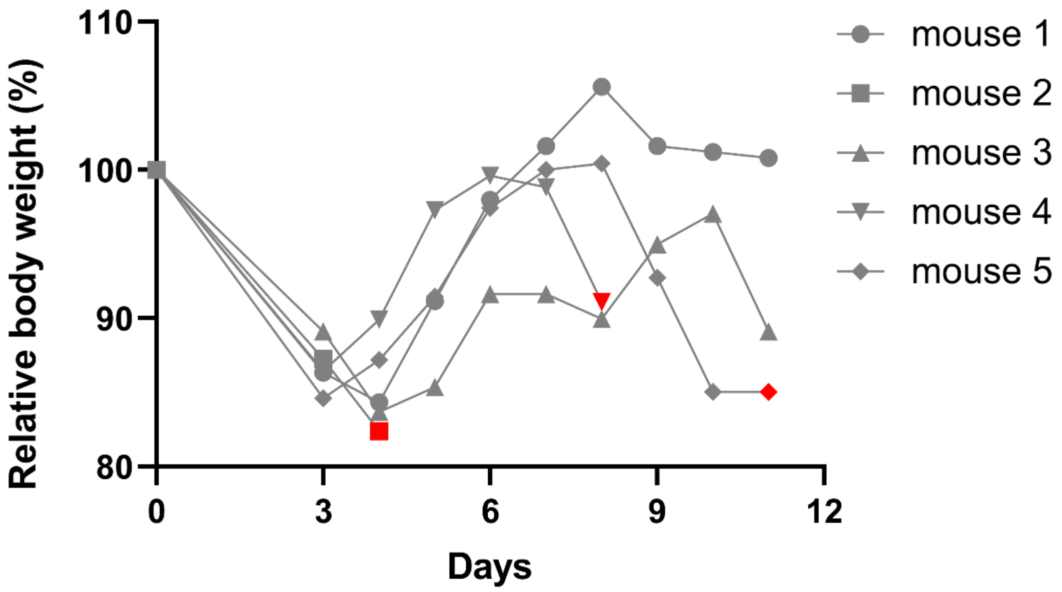

Supplementary Figure S1. Body weight of female antibiotic-treated *Cyp2c70*$^{-/-}$ mice shown as a percentage of the body weight before antibiotics. The red symbols indicate the moments of application of humane endpoints due to severe loss of body weight and/or lethargy.

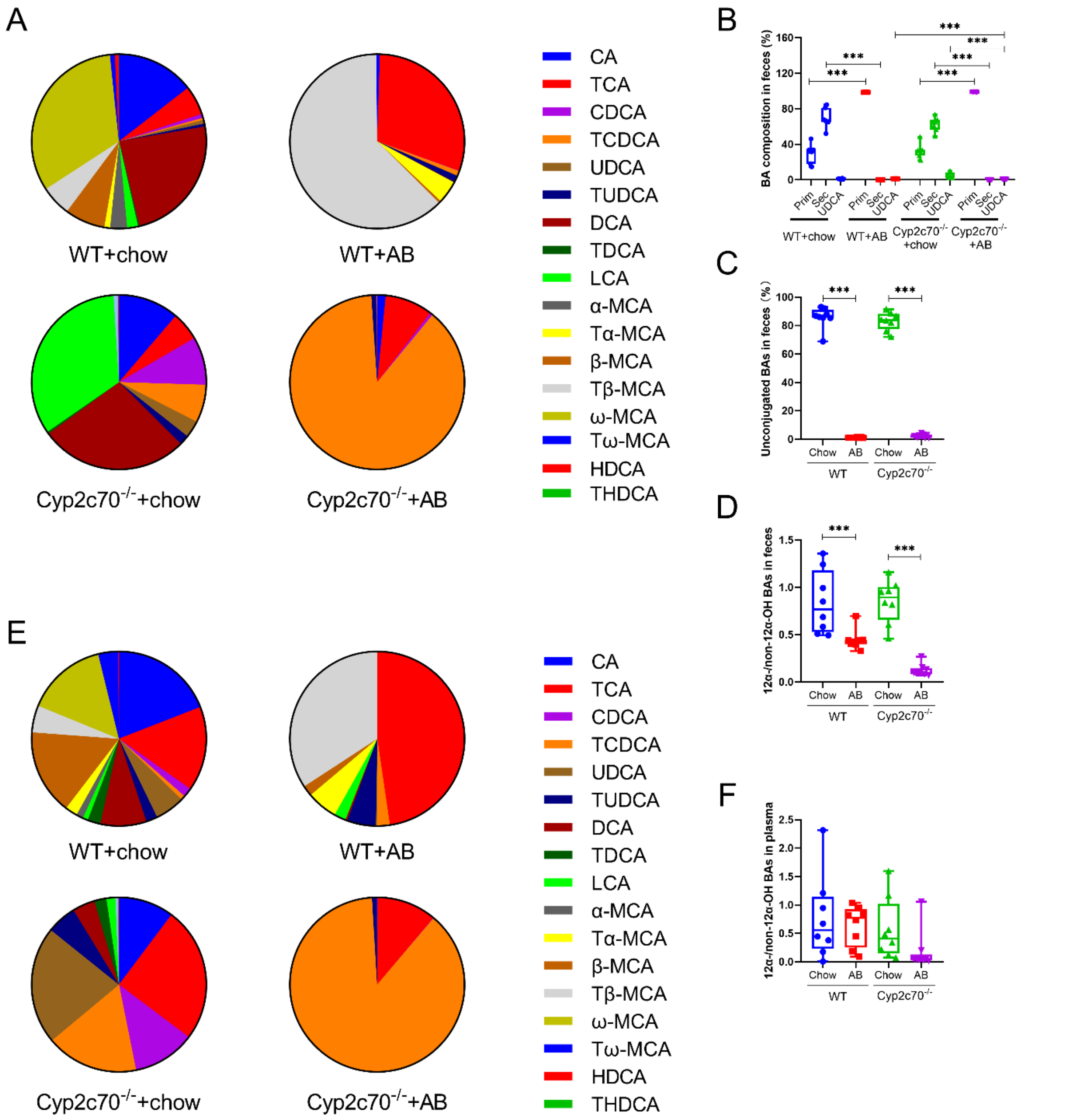

Supplementary Figure S2. Bile acid profiles in feces and plasma of male WT and *Cyp2c70*$^{-/-}$ mice fed a regular chow diet or an antibiotics-containing chow diet. (A) BA profiles in feces. (B) Percentage of secondary BAs in feces.Primary BAs: cholic acid, chenodeoxycholic acid, α/β-muricholic acid and conjugates. Secondary BAs: deoxycholic acid, lithocholic acid, hyo(deoxy)cholic acid, ω-muricholic acid and conjugates.(C) Percentage of unconjugated BAs in feces. (D) 12α-/non-12α-OH BA ratios in feces. (E) BA profiles in plasma. (F) 12α-/non-12α-OH BA ratios in plasma. *** p<0.001 according to Kruskal-Wallis H test followed by Conover post hoc comparisons. AB, antibiotics; BA, bile acid.

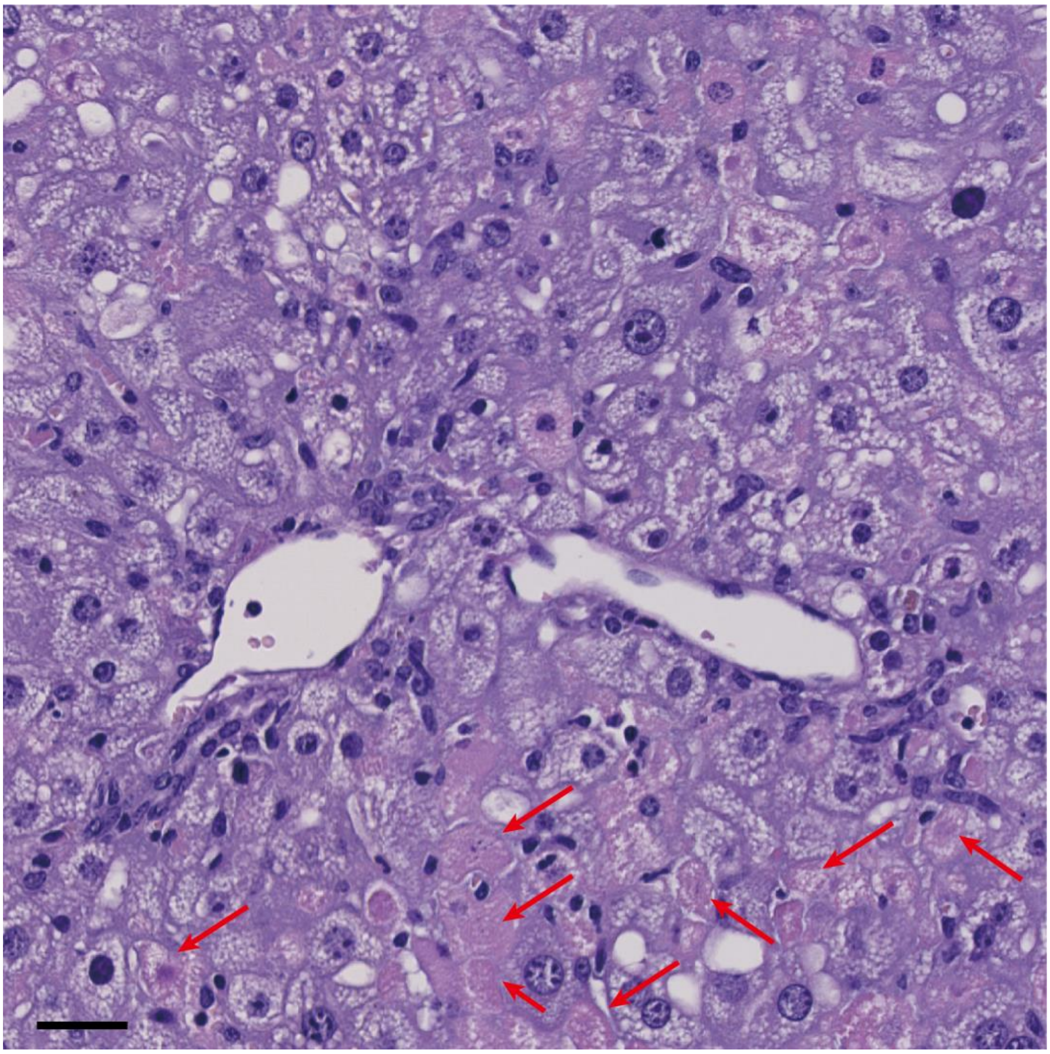

Supplementary Figure S3: Frequent hepatocyte cell death in male Cyp2c70$^{-/-}$ mice upon antibiotics treatment. Representative image of liver section stained with hematoxylin and eosin (H&E) (bar represents 70 µm). The red arrows indicate dead cells.

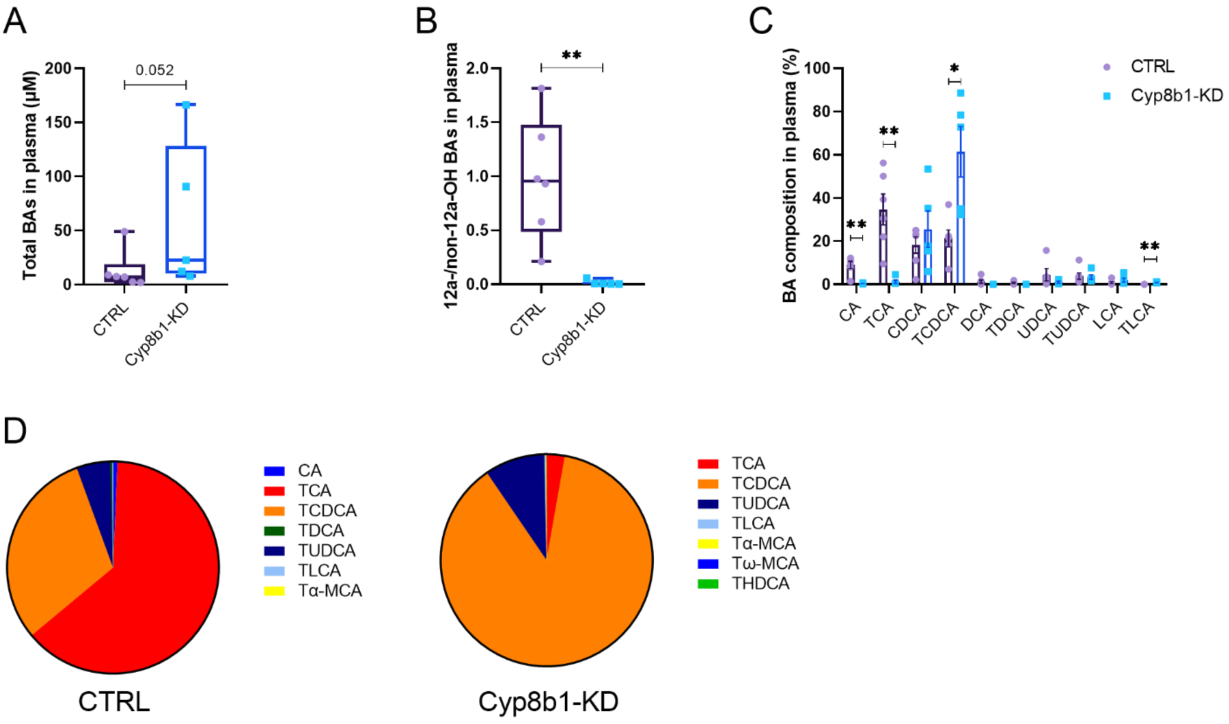

Supplementary FigureS4. Bile acids in plasma and gallbladder bile of male *Cyp8b1*-KD mice and controls. (A) Total BAs in plasma. (B) 12*α*-/non-12*α*-OH BA ratios in plasma. (C) BA composition in plasma. (D) BA profiles in gallbladder bile. * p<0.05, ** p<0.01 as determined by Mann-Whitney U test. BA, bile acid.

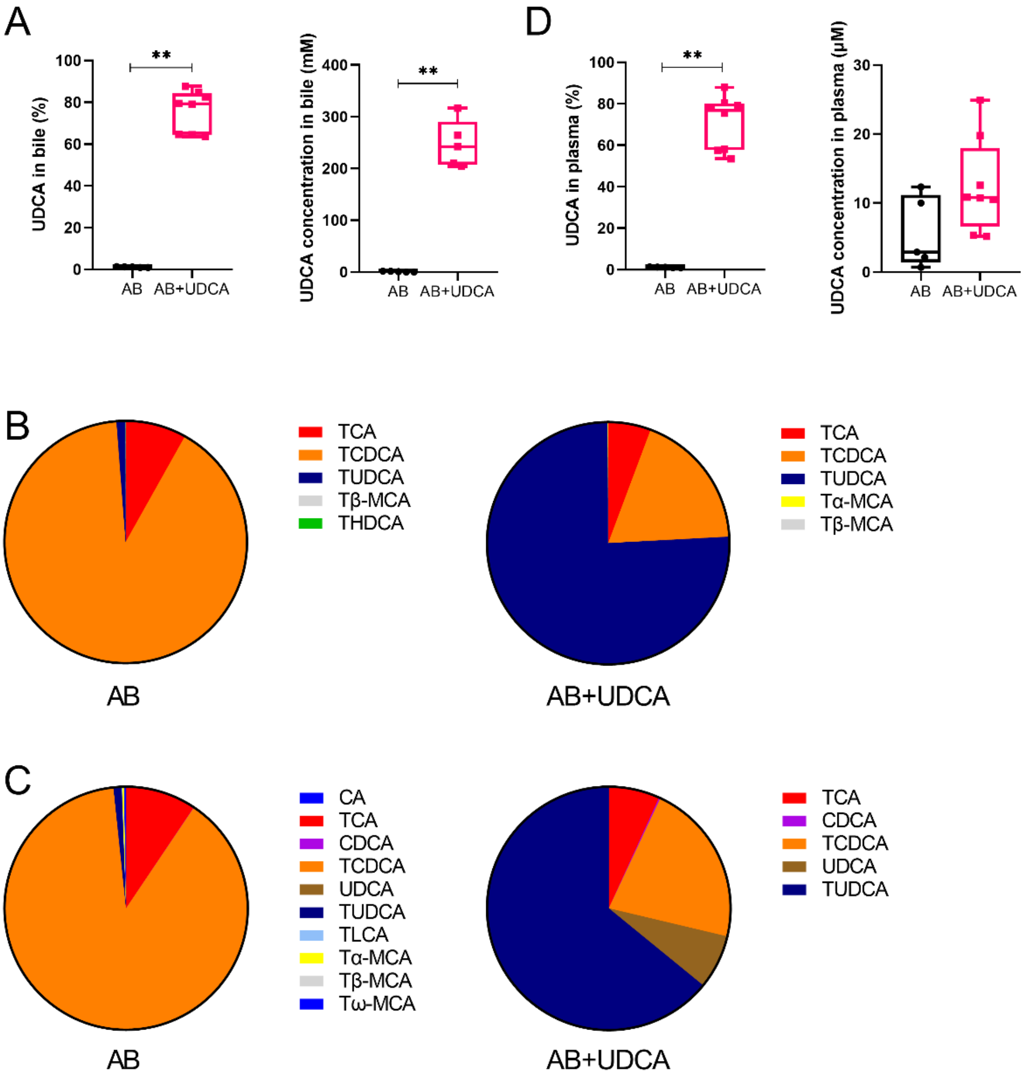

Supplementary Figure S5. Bile acids in gallbladder bile and plasma of female Cyp2c70$^{-/-}$ mice fed a diet containing antibiotics or antibiotics + UDCA. (A) Percentage of total BAs and concentration of UDCA in gallbladder bile. (B) BA profiles in gallbladder bile. (C) BA profiles in plasma. (D) Percentage of total BAs and concentration of UDCA in plasma. ** p<0.01 as determined by with Mann-Whitney U test. AB, antibiotics; BA, bile acid.
